# In vivo engraftment into the cornea endothelium using extracellular matrix shrink-wrapped cells

**DOI:** 10.1038/s43246-022-00247-1

**Published:** 2022-04-26

**Authors:** Rachelle N. Palchesko, Yiqin Du, Moira L. Geary, Santiago Carrasquilla, Daniel J. Shiwarski, Irona Khandaker, James L. Funderburgh, Adam W. Feinberg

**Affiliations:** 1 Department of Biomedical Engineering, Carnegie Mellon University, Pittsburgh, PA 15213, USA.; 2 Department of Ophthalmology, University of Pittsburgh, Pittsburgh, PA 15213, USA.; 3 Department of Materials Science & Engineering, Carnegie Mellon University, Pittsburgh, PA 15213, USA.

## Abstract

Cell injection is a common clinical approach for therapeutic delivery into diseased and damaged tissues in order to achieve regeneration. However, cell retention, viability, and engraftment at the injection site have generally been poor, driving the need for improved approaches. Here, we developed a technique to shrink-wrap micropatterned islands of corneal endothelial cells in a basement membrane-like layer of extracellular matrix that enables the cells to maintain their cell-cell junctions and cytoskeletal structure while in suspension. These μMonolayers exhibited the ability to rapidly engraft into intact, high-density corneal endothelial monolayers in both in vitro and in vivo model systems. Importantly, the engrafted μMonolayers increased local cell density, something that the clinical-standard single cells in suspension failed to do. These results show that shrink-wrapping cells in extracellular matrix dramatically improves engraftment and provides a potential alternative to cornea transplant when low endothelial cell density is the cause of corneal blindness.

Organ and tissue transplants are the only options for patients with end-stage organ failure, and while effective, >1 million people are unable to benefit due to global donor shortages^1^. Patients must also remain on immunosuppressants for life with major side effects, experience a high rate of organ failure and rejection, and have no access to transplantation in many parts of the world^2^. As an alternative, cell-based therapies have long been thought of as a potential therapeutic option for a range of diseases and injuries caused by tissue and organ failure such as myocardial infarction^3^, diabetes^4,5^, corneal blindness^6^, and cystic fibrosis^7,8^. The goal is to deliver viable cells that integrate into the target tissue and replace damaged or dysfunctional cells in order to stop or reverse disease progression. Potential advantages compared to transplant include minimally invasive cell delivery without the need for extensive surgery, use of autologous cells to avoid immune rejection, and the ability to improve tissue and organ function earlier in the disease process and thus entirely avoiding end-stage failure. Indeed, the past decade has seen advances in research and development of cell-based therapies based on autologous adult stem cells and induced pluripotent stem (iPS) cells^9–11^. However, a simple injection of cells into tissues has shown only limited clinical success in many applications due to low cell viability after injection as well as poor retention at the injection site and engraftment into the damaged tissue^10,12,13^. Thus, there remains a critical need for new technologies that can improve cell delivery, engraftment, and function.

The cornea serves as a clinically relevant tissue for the development of new cell delivery approaches because at >50,000 procedures annually in the US, it is transplanted more than all other solid organs combined^14^. Specifically, here we are focused on the corneal endothelium (CE), a single layer of cells that lines the posterior surface of the cornea and is responsible for maintaining proper corneal thickness and clarity through the regulation of stroma hydration. Nearly 50% of all corneal transplants are due to failure of the CE, primarily due to loss of CE cells that are cell cycle arrested and cannot replicate to repair damage or injury^15–18^. This subsequently leads to failure to properly pump fluid from the stroma to the aqueous humor once the cell density drops below ~500 cells/mm^2^, resulting in corneal edema and clouding^19,20^. Current clinical treatment for CE failure is fullthickness penetrating keratoplasty (PK) or partial-thickness transplants such as Descemet membrane endothelial keratoplasty (DMEK) and Descemet stripping automated endothelial keratoplasty (DSAEK)^14,21^. These lamellar techniques have shown improvement over PK, with evidence that immune rejection is reduced with less stroma and extracellular matrix (ECM) transplanted^22,23^. Further, the eye is considered to be immune-privileged and eye drops are usually adequate rather than systemic immunosuppression. However, chronic rejection and limited donor supply in many parts of the world have motivated the development of new methods to inject CE cells into the anterior chamber to repopulate the endothelium and restore function^6^. The problem these cell therapies have faced in the eye is the same as in other tissues and organs, which is effective delivery and engraftment^10,12,13^. In fact, most approaches require the existing CE to be removed through scraping or cryogenic injury of the cornea in order to provide a place for the delivered cells to attach^6^.

Here we report the development of a new cell delivery method designed to enhance cell attachment and engraftment into tissues in vivo without requiring any induced damage to achieve integration. The challenge to delivering cells to the CE, and to epithelial and endothelial layers in general, is that these tissues are characterized by robust cell–cell injunctions and in general have evolved to act as barriers to keep things out. Thus, it has proved challenging to deliver single cells in suspension, which do not have a mechanism to attach and integrate. To address this, we hypothesized that small patches of CE with intact tight junctions and cytoskeletal structure may exhibit improved adhesion and integration into existing CE monolayers compared to single cells. Specifically, our goal was to create a method where we could deliver viable cells to intact CE monolayers without removal of any cells and achieve integration that would increase cell density. To do this we developed an approach to shrink-wrap micronscale monolayers (μMonolayers) of CE cells within an engineered layer of ECM using an adaptation of our previously reported surface-initiated assembly technique^24,25^. This technology enables the cells within the μMonolayers to maintain high viability, tight junctions, and cytoskeletal structure throughout the release and injection process. Most importantly, the μMonolayers are able to integrate into existing CE monolayers and significantly increase cell density in both in vitro and in vivo assays. These results suggest that this technique could be used to increase cell density to treat corneal blindness without requiring the removal of the existing CE and enhance the engraftment of injected cells.

## Results

### Shrink-wrapped CE cell μMonolayers maintain cytoskeletal structure, tight junctions, and high viability.

**Fig. 1:**
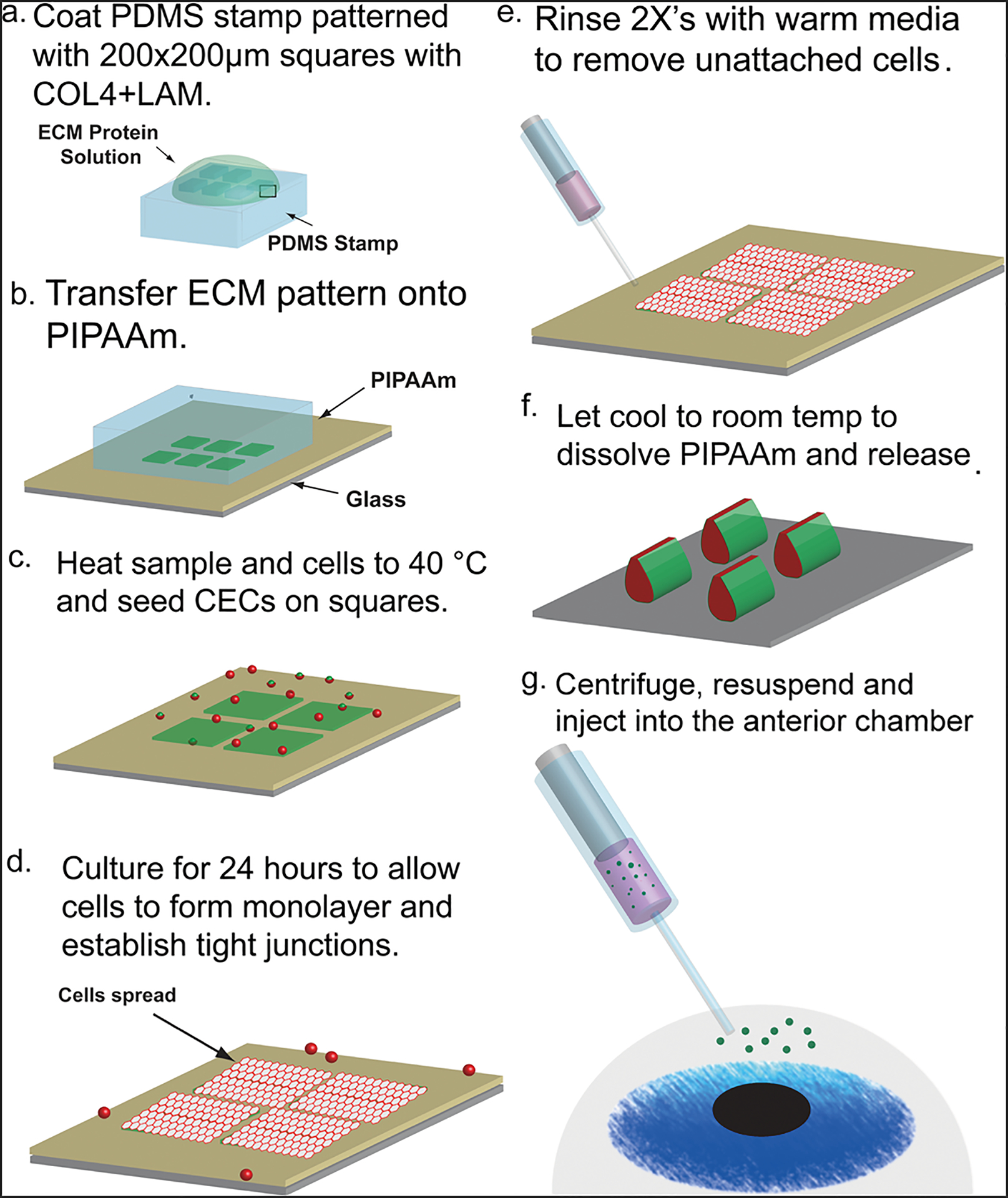
Schematic representation of the process for shrink-wrapping and injecting corneal endothelial cell μMonolayers. Steps **a**, **b** Surface-initiated assembly techniques are used to engineer 200 μm square, 5 nm-thick ECM scaffolds on the thermoresponsive polymer, PIPAAm. Steps **c**, **d** The samples and cells are then heated to 40 °C before seeding the cells on the squares and culturing for 24 hours. Steps **e**, **f** After 24 hours, samples are rinsed with warm media and cooled to room temperature to trigger the dissolution of the PIPAAm and shrink-wrapping/release of the μMonolayers of corneal endothelial cells before injection into the anterior chamber of the eye (Step **g**).

To engineer the CE cell μMonolayers, bovine or rabbit CE cells were seeded onto micropatterned 200 × 200 μm squares of ECM proteins (1:1 laminin and collagen type IV) that are ~5 nm thick (Supplementary Fig. 1) and were fabricated via surface-initiated assembly on thermoresponsive poly(n-isopropylacrylamide) (PIPAAm) substrates (Fig. 1). Laminin (LAM) and collagen IV (COL4) were chosen because they are two of the main components in basement membranes, specifically Descemet’s membrane, of the CE. Additionally, our previous work has shown that bovine CE cells grow best on a mixture of these two proteins^26,27^. Bovine CE cells were chosen as they can be isolated in large quantities from animals of a defined age and proliferate readily in vitro, providing the consistent phenotypic behavior needed for the in vitro studies. Rabbit CE cells were isolated for all ex vivo and in vivo studies in rabbit eyes to provide an allogeneic cell source and eliminate any xenogeneic effects. The cells were cultured on the scaffolds for 24 hours, which was the minimum found to allow the cells to adhere to the ECM square, establish cytoskeletal structure and tight junctions, and form a confluent layer. At greater than 48 hours, the PIPAAm becomes less stable and occasionally the μMonolayers can be prematurely released. Upon thermally triggered dissolution of the PIPAAm, the ECM square releases from the surface, and as shown in our previously published papers on surface-initiated assembly, the ECM contracts in the x and y directions^25^. This contraction is due to pre-stress in the patterned ECM and the CE cells from being spread on the PIPAAm surface, and once released the μMonolayers (both the CE cells and the ECM) fold inward and are small enough to be injected through a small gauge needle. The ECM remains attached to the basal side of the μMonolayer during and after the shrink-wrapping process, suggesting that the apical-basal polarity of the CE cells is maintained.

**Fig. 2:**
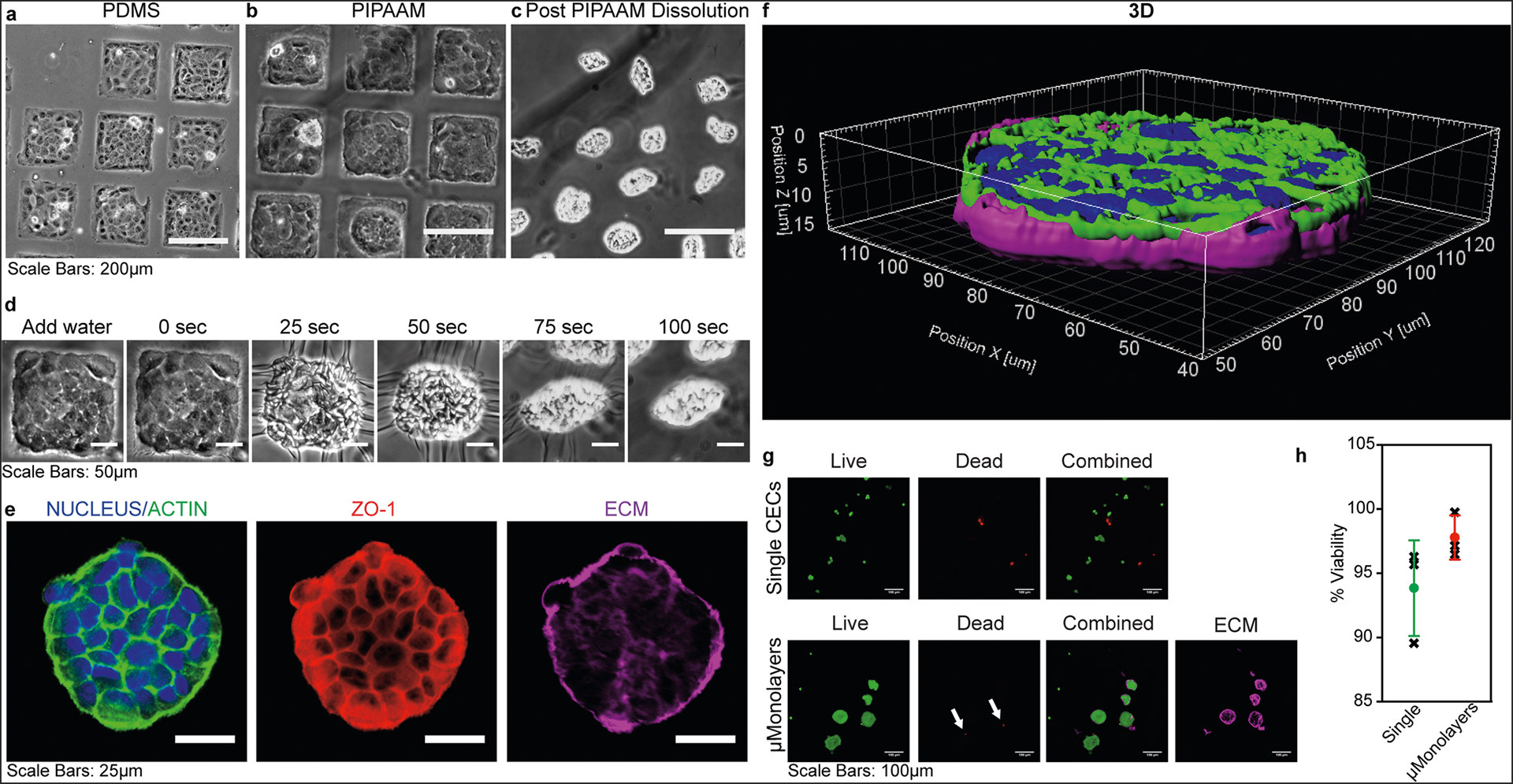
Bovine CE cells form μMonolayers on ECM squares and maintain their structure and viability through shrink-wrapping and injection. **a** CE cells form monolayers on ECM squares microcontact printed onto PDMS (used as a control) and **b** on the thermoresponsive polymer PIPAAm. **c** Once the PIPAAm is dissolved the CE cell μMonolayers contract and are shrink-wrapped in the ECM squares. **d** The release and shrink-wrapping of μMonolayers occurs quickly, in <100 seconds once the water + sample cools to room temperature. **e** Confocal microscopy images show that after injection, the CE cells maintain both their cytoskeletal structure (F-actin, green), tight junctions (ZO-1, red), and adherence to the ECM scaffold (LAM + COL4, purple). **f** A 3D render of a shrink-wrapped CE cell μMonolayer 30 minutes after injection onto a glass surface illustrating how it begins to relax and return to its original shape. **g** Representative live/dead images of control single CE cells and shrink-wrapped CE cells show that both types of cells are viable with very few dead cells present. **h** Live/dead data showed no significant difference in viability between single cells (93 ± 4 %) and shrink-wrapped cells (97 ± 2%) following injection through a 28 G needle (n = 3 biologically independent samples for each; mean ± stdev represented by the dots and bars, individual samples are shown as the black x’s; N.S. by Student’s t test Single vs. μMonolayers).

To create the μMonolayers, we needed to ensure that the CE cells would adhere and spread on the ECM squares to form confluent patches and then properly shrink-wrap. As a control, bovine CE cells were seeded onto ECM squares micropatterned onto polydimethylsiloxane (PDMS) substrates because it is not temperature sensitive, and previous studies have established cell growth on this surface^25,28–31^. After 24 hours, the CE cells on the ECM squares on PDMS were adhered and spread into a monolayer as expected (Fig. 2a). Next, we repeated this by seeding CE cells on ECM squares patterned on PIPAAm, and the cells exhibited a similar morphology when viewed under phase microscopy (Fig. 2b). Upon dissolution of the PIPAAm and thermal release, the cells remained interconnected and were successfully shrink-wrapped within the ECM squares into μMonolayers (Fig. 2c). Time-lapse images show that once the media reached room temperature and the PIPAAm dissolved (0 sec), the shrink-wrapping process occurred quickly in <100 seconds (Fig. 2d, Supplementary Video 1).

After release, the shrink-wrapped μMonolayers were collected, centrifuged, injected through a 28 G needle onto a glass coverslip, and then allowed to settle for 30 min before staining to investigate the morphology, structure, and viability of the CE cells. The shrink-wrapped CE cells exhibited continuous ZO-1 at the borders and a cortical F-actin structure indicating that the cells maintained their tight junctions and cytoskeletal structure throughout the release and injection process (Fig. 2e). Immediately post-release, the shrink-wrapped μMonolayers contracted tightly into small clusters (Fig. 2c), however, ~30 minutes after release and injection the μMonolayers relaxed and returned to a disc-like morphology as they settled onto the surface (Fig. 2f). While patterned as a square, the μMonolayers always adopt a disc-like morphology after injection likely due to the cell generated pre-stress prior to release and energy minimize post-release. The ECM uniformly covers the basal side of the μMonolayer, covering ~50% of the surface area (Supplementary Fig. 2). This establishes that the shrink-wrapping process and subsequent injection through a small gauge needle do not disrupt cell–cell adhesions or cause damage to the CE cells in the μMonolayer, both of which are important to the function of CE cells. High cell viability in the μMonolayers after injection was confirmed using a Live/Dead cytotoxicity assay and compared to enzymatically released single cells (Fig. 2g). Confocal microscopy images revealed that the only dead cells were those that were not integrated into the shrink-wrapped μMonolayers, with cells in the μMonolayers showing very high viability (Fig. 2g, shown by the arrows). Quantitative image analysis showed that single CE cells in suspension had 93 ± 4% viability and the CE cells within the shrink-wrapped μMonolayers had 97 ± 2% viability, though this difference was not statistically significant (Fig. 2h).

### Shrink-wrapped μMonolayers rapidly adhere and spread to form a CE monolayer on collagen gels in vitro.

**Fig. 3:**
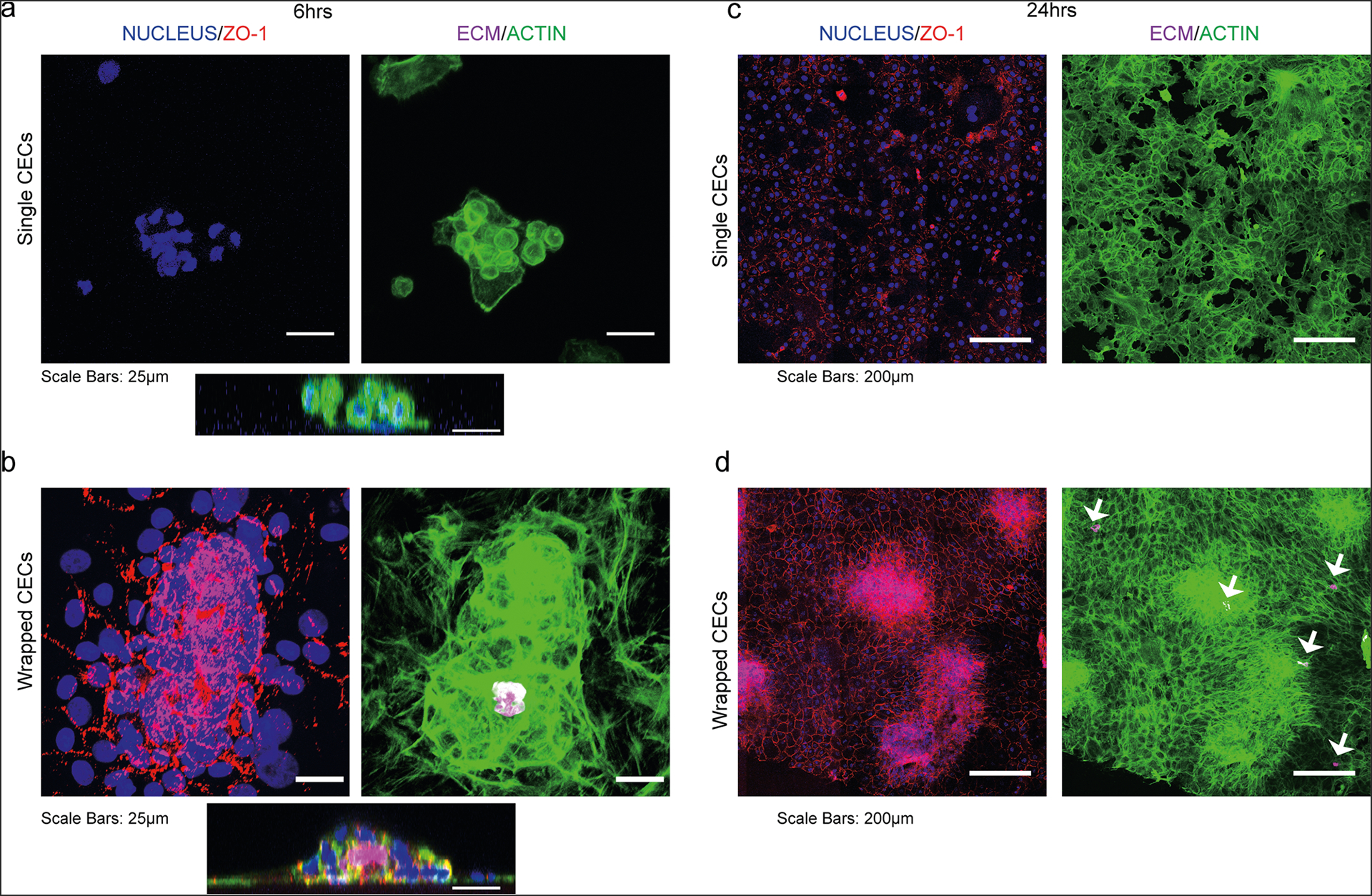
Shrink-wrapped bovine CE cell μMonolayers maintain ZO-1 expression and F-actin cytoskeleton as they grow out of the ECM scaffolds to form a monolayer on a collagen type I stromal mimic. **a** Six hours after reseeding onto a collagen type I gel, the single CE cells have no established F-actin cytoskeleton or ZO-1 expression. The cross-sectional view shows the rounded cell morphology. **b** In contrast, the CE cells in the shrink-wrapped μMonolayers have maintained their ZO-1 expression and F-actin cytoskeleton, while growing out of the ECM scaffolds. The cells at the periphery of the shrink-wrapped CE cells are also expressing ZO-1. The cross-sectional view shows that the cells are spreading. **c** At 24 hours, single CE cells have begun to spread and cover almost the entire scaffold. **d** At 24 hours, the CE cells have already grown out of the ECM scaffolds and formed an almost complete monolayer. For **a**–**d**: Nucleus = blue, ZO-1 = red, ECM(COL4) = magenta, F-actin = green.

To assess the potential of the shrink-wrapped μMonolayers for cell injection therapy, we first performed an in vitro assay using a compressed collagen type I gel as a model of a denuded corneal stroma. Shrink-wrapped bovine μMonolayers or single CE cells in suspension (as the control) were seeded onto compressed collagen type I gels by delivery through a 30-gauge needle. Samples were fixed and stained at 6 hours to observe initial attachment and adhesion and at 24 hours to observe spreading and outgrowth. At 6 hours post injection, single CE cells were mostly rounded with very little spreading observed (Fig. 3a) and the F-actin staining showed no actin filaments, additionally, there was no ZO-1 observed. In contrast, the CE cells from the shrink-wrapped μMonolayers maintained their cytoskeletal structure and tight junctions, as evidenced by the F-actin filaments and continuous ZO-1 expression at the cell borders (Fig. 3b). Examining the samples using 3D confocal imaging confirmed that single CE cells were rounded and had few contacts between cells (Fig. 3a) whereas the shrink-wrapped μMonolayers had reoriented with cells directly attached to the collagen and the ECM scaffolds now present within the center of the monolayer (Fig. 3b). After 24 hours, the single CE cells covered most of the collagen substrate and had a more defined cytoskeletal structure (Fig. 3c) but with many F-actin stress fibers across the cell bodies rather than being primarily cortical. Additionally, the single CE cells exhibited very low ZO-1 staining, expressed discontinuously at the cell borders (Fig. 3c). In contrast, the shrink-wrapped μMonolayers had continuous ZO-1 at all cell borders and abundant cortical F-actin (Fig. 3d), which closely resembled the structure of in vivo CE cells^26^. The remnants of the ECM squares used for shrink-wrapping were visible as indicated by the arrows in Fig. 3d. These results show that CE cells in shrink-wrapped μMonolayers more rapidly repopulate a collagen substrate than single CE cells over the first 24 hours of culture.

### Shrink-wrapped μMonolayers show enhanced engraftment into existing CE monolayers and increase CE density in vitro.

**Fig. 4:**
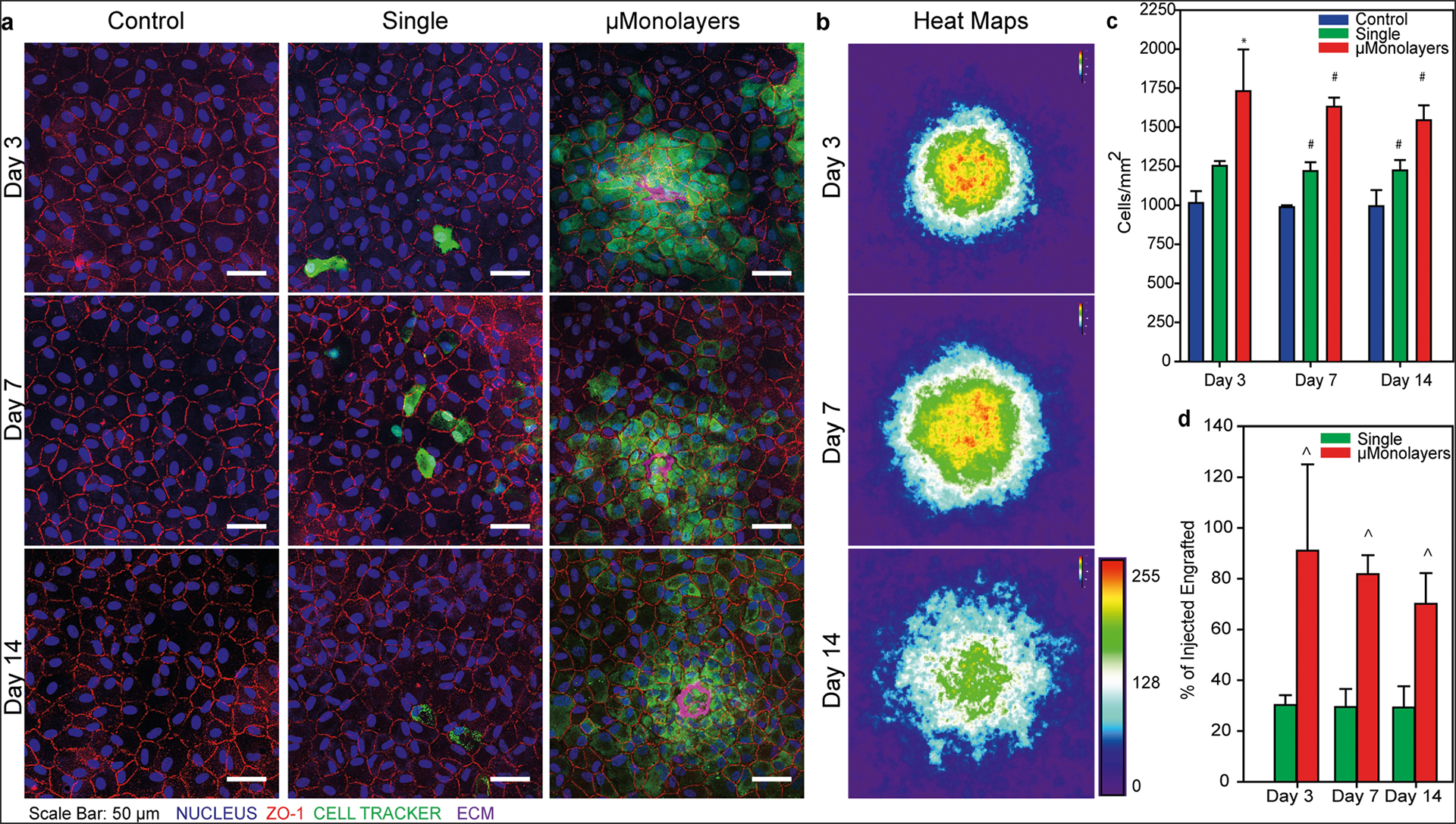
Injected shrink-wrapped bovine μMonolayers integrate into existing monolayers of bovine CE cells and significantly increase the density compared to single bovine CE cells. **a** Cell Tracker labeled single cells and shrink-wrapped cells were visible at all time points however, significantly more shrink-wrapped cells were present at all time points and the ECM scaffolds were still visible 14 days after injection. Scale bars = 50 μm. **b** Heat maps of Cell Tracker positive pixels show that the cells in the shrink-wrapped μMonolayers initially integrate into a tight cluster and then the density equilibrates as the cells spread out slightly (Day 3 n = 33 independent shrink-wrapped clusters, Day 7 n = 37, Day 14 n = 40, scale bar is arbitrary units). **c** The cell density of the monolayers was calculated and compared on days 3 (n = 4 biologically independent samples for each sample type, each n is an average of 10 images per sample), 7 (control n = 3, single and μMonolayer n = 4, biologically independent samples, each n is an average of 10 images per sample) and 14 (single n = 3, control and μMonolayer n = 4, biologically independent samples, each n is an average of 10 images per sample). Data are represented as mean ± std dev and compared using one-way ANOVA on ranks with Tukey’s test (day 3) or one-way ANOVA with Tukey’s Test (day 7 and 14), where * (p < 0.05) indicates statistically significantly different from control and ^#^ (p < 0.05) indicates statistically significantly different from all other samples. **d** The percentage of the injected single and shrink-wrapped cells that were integrated into the monolayers was calculated on days 3 (n = 4 biologically independent samples for each sample type), 7 (control n = 3, single and μMonolayer n = 4, biologically independent samples) and 14 (single n = 3, control and μMonolayer n = 4, biologically independent samples) (each n is an average of 10 images per sample). Data are represented as mean ± std dev and compared using t test at each time point, where ^ (p <0.05) indicates a statistically significantly different from single cells.

There are currently clinical trials in Japan using human iPS-derived CE cells to restore the endothelium, but it requires removal of the existing CE cells in order to make space to deliver the new cells and allow them to attach^6,32^. This is because cells seeded onto an existing endothelial or epithelial layer typically show very low attachment and engraftment. Previous studies have shown that cell–cell and cell-ECM contacts are important for the survival and function of endothelial cells^33–35^; therefore, we hypothesized that the shrink-wrapped μMonolayers would be able to engraftment into existing CE monolayers through enhanced cell–cell and cell-ECM binding. To test this, we seeded shrink-wrapped bovine CE cell μMonolayers in vitro onto a low-density bovine CE monolayer to mimic the low CE density in patients that need a cornea transplant. The CE cells were labeled with CellTracker Green and μMonolayers or single-cell controls and were delivered through a 30 G needle to seed the samples. CE monolayers that were not seeded with any additional cells served as negative controls. The seeded CE single cells and μMonolayers were allowed to settle onto the samples for 3 hours before rinsing and adding fresh media to mimic the procedure used for clinical CE cell injection in animal models and human patients, where they remain face down for 3 hours post injection^6,33,36^. Samples were then cultured for 3, 7, and 14 days and analyzed for engraftment (Fig. 4a). At each time point, very few single CE cells were integrated into the CE monolayers, and large areas of the samples had to be imaged to find even a few labeled cells. In contrast, the shrink-wrapped μMonolayers were well-integrated on day 3 and appeared more densely packed compared to the original monolayer. On day 7, the shrink-wrapped CE cells had completely integrated into the existing CE monolayer, still appearing more densely packed but also more spread out from the attachment site (Fig. 4a). By day 14, the μMonolayers appeared to be well engrafted and the density of the newly integrated CE cells had equilibrated with the CE cells in the existing monolayer, achieving an increased overall cell density.

To determine how well the shrink-wrapped CE cell μMonolayers engrafted and increased the cell density of the low-density CE monolayers, the density was analyzed at each time point. Overall, the single CE cells increased the monolayer density ~20% while the shrink-wrapped CE cell μMonolayers increased the monolayer density ~50%, even though the same total number of cells were seeded for each condition (Fig. 4c). On day 3, the shrink-wrapped μMonolayers significantly increased cell density compared to controls and on days 7 and 14 the shrink-wrapped μMonolayers significantly increased cell density compared to both controls and samples seeded with single CE cells. These results establish that the shrink-wrapped μMonolayers adhere, engraft, and then spread out into an existing CE monolayer in a manner that single CE cells cannot. Further, we generated heat maps using images of the CellTracker green-labeled CE cells in the shrink-wrapped μMonolayers to confirm that the cells were spreading out over time (Fig. 4b). The results confirm that on day 3 the CE cells are contained in a small area and that over time they spread out into the surrounding monolayer and equilibrate in density. This makes sense because even though the images of fixed CE monolayers make it look like the cells are stationary, time-lapse images routinely show that cells are constantly moving within epithelial layers^37,38^, which should facilitate the spreading out of the shrink-wrapped CE cells. This equilibration process also explains the perceived decrease in cell density from day 3 to 14, where the cell density overall is not decreasing, it is just becoming more homogenous across the CE monolayer. This was confirmed by running a one-way analysis of variance (ANOVA) within each sample type comparing the densities over time, which showed that the time point had no statistically significant effect. Further, we quantified the percentage of cells that engrafted for both the single cells and shrink-wrapped cells (Fig. 4d) and found that at each time point ~30% of seeded single cells engrafted while a statistically significant ~75–90% of shrink-wrapped cells engrafted.

### Shrink-wrapped μMonolayers adhere and begin to integrate into the CE within 3 hours.

**Fig. 5:**
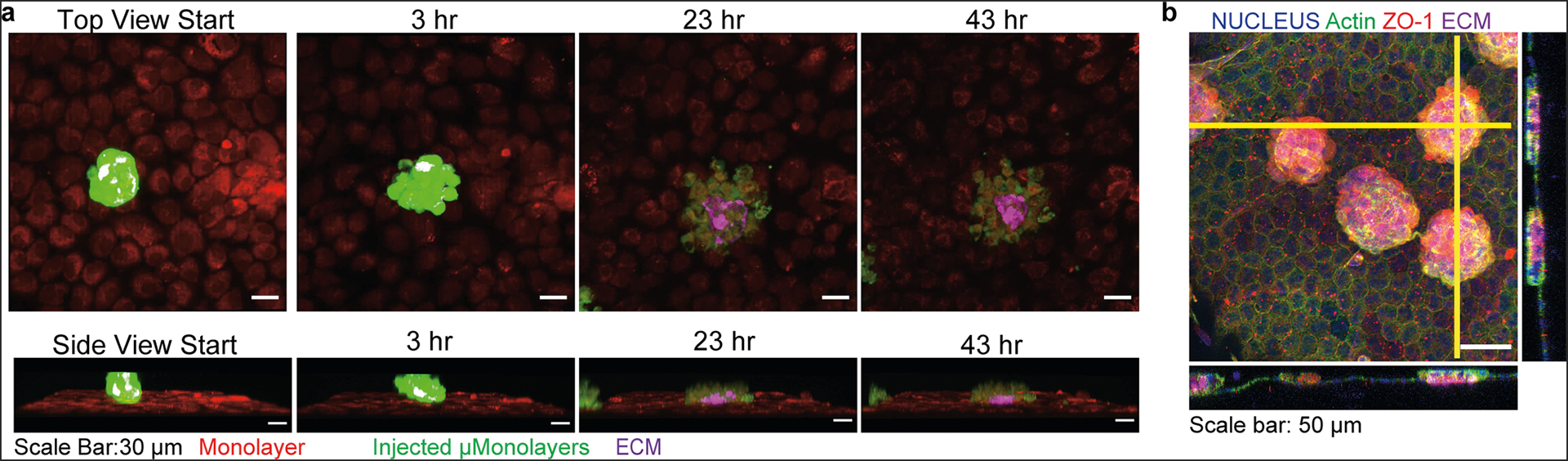
Shrink-wrapped μMonolayers begin to integrate into low-density CE monolayers and ex vivo corneas within 3 hours. **a** Time-lapse images from live confocal imaging of the integration of shrink-wrapped bovine μMonolayers (Cell Tracker green) into an engineered bovine CE monolayer (Cell Tracker Orange). At 3 hours, the μMonolayers have attached and begun to integrate and by 43 hours the cells are almost completely integrated into the monolayer. **b** Confocal images show that the shrink-wrapped rabbit μMonolayers had begun to integrate into the ex vivo rabbit CE and the ECM scaffold is observed to be between μMonolayers and the existing rabbit CE. The yellow vertical and horizontal lines indicate the places at which the orthogonal views were obtained.

Clinical injection of single CE cells requires that patients lie face down for 3 hours post injection to allow for cell attachment to the CE on the posterior of a denuded cornea^6,32,33^. Thus, a major question for the shrink-wrapped μMonolayers is how they achieve improved engraftment and how long it takes compared to single cells. To address this, we performed live confocal imaging of engraftment in vitro by labeling the shrink-wrapped bovine μMonolayers with CellTracker^™^ Green and the cells in the low-density monolayer with CellTracker^™^ Orange. Note that we performed this study in vitro because we could take time-lapse confocal images, which would not be feasible in an animal model. By collecting a Z-stack every hour for 48 hours, we were able to clearly observe the dynamics of the integration process from both top-down and side views (Supplementary Videos 2 and 3). At 3 hours post injection, the shrink-wrapped μMonolayers had begun to attach and flatten on top of the low-density monolayer (Fig. 5a). Over time the shrink-wrapped μMonolayers continued to flatten and move around as the cells moved into the underlying low-density monolayer. By 43 hours, the shrink-wrapped μMonolayer was almost completely integrated into the CE monolayer and appeared to be in the same imaging plane as the low-density monolayer and not sitting on top of it. Initially, the ECM square used in the shrink-wrapping process is on the basal side of the μMonolayer and thus touches the apical side of the low-density monolayer. As engraftment proceeds the ECM square appears to detach as the cells migrate out into the monolayer but remains centrally located at the location where the μMonolayers initially attached, which is consistent with results from the fixed time point experiments (Fig. 4a).

Although the time-lapse imaging results suggested that 3 hours is sufficient for the shrink-wrapped μMonolayers to attach to the CE, potential differences between in vitro and in vivo conditions caused us to assess attachment to the native cornea. To do this, we switched to rabbit CE cells and injected shrink-wrapped μMonolayers ex vivo into the anterior chamber of enucleated rabbit eyes. The eyes were then incubated with the cornea facing down for 3 hours to allow attachment of the shrink-wrapped μMonolayers before fixation and staining of the whole globe (Supplementary Fig. 3). Confocal imaging of the corneas showed numerous shrink-wrapped μMonolayers attached over the entirety of the posterior surface (Fig. 5b). At this 3-hour time point, the shrink-wrapped μMonolayers were still disc-like in shape and oriented with the ECM layer facing the CE on the posterior surface of the cornea. This is consistent with the observations from the time-lapse experiments. These experiments provided confidence that 3 hours was sufficient for gravitational settlement and attachment of the shrink-wrapped μMonolayers for subsequent in vivo experiments.

### Shrink-wrapped μMonolayers show robust engraft into the CE in vivo.

**Fig. 6:**
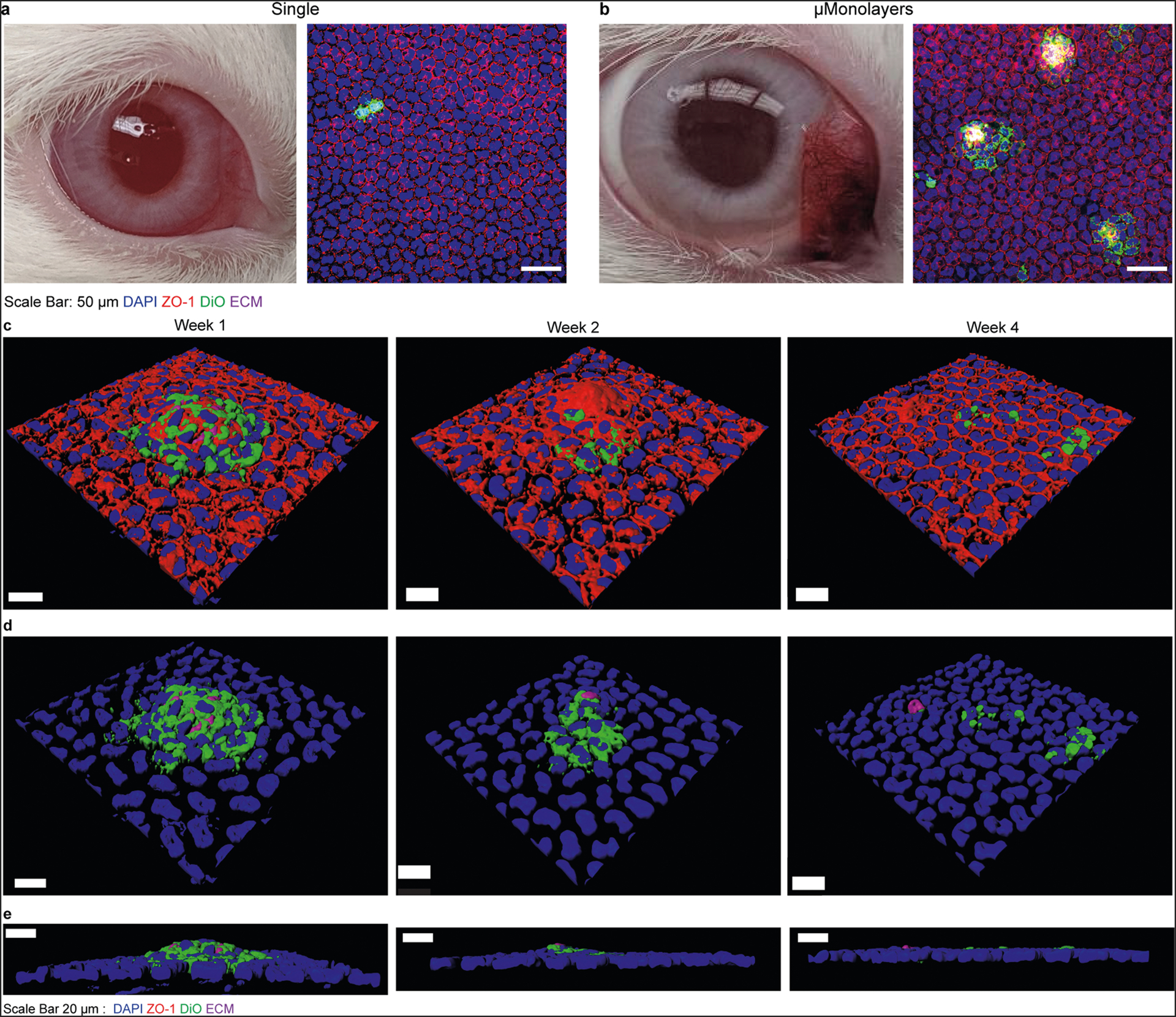
Shrink-wrapped μMonolayers integrate into the existing healthy rabbit CE. **a** Rabbit corneas injected with single cells remained clear at 1 week post injection. However, very few DiO-labeled single cells were observed integrated into the rabbit CE. **b** The rabbit cornea injected with μMonolayers also remained clear 1-week post injection and numerous clusters of μMonolayers were observed in each cornea with the continuous ZO-1 at the borders between DiO-labeled cells and native rabbit CE cells. **c** Confocal microscopy images showing the integrated DiO-labeled shrink-wrapped μMonolayers at 1, 2-, and 4 weeks post injection. Nuclei = blue, DiO-labeled cells (green), ZO-1 (red), ECM nanoscaffold (purple). **d** The same images from **c** with the ZO-1 removed to highlight the nuclei of both the healthy rabbit endothelium and injected cells (blue), DiO-labeled injected rabbit cells (green), and ECM nanoscaffold (purple) from the shrink-wrapping process at 1, 2-, and 4 weeks post injection. **e** The orthogonal views of the confocal images shown in **d** showing the integration of the shrink-wrapped μMonolayers into the healthy rabbit endothelium over the 4-week period post injection. Scale bars in **a** and **b** are 50 μm, and in **c**–**e** are 20 μm.

Having demonstrated enhanced engraftment of the shrink-wrapped μMonolayers in vitro and ex vivo, we moved next to an in vivo rabbit model. First, we assessed the basic feasibility of injecting the shrink-wrapped rabbit μMonolayers in vivo and achieving engraftment. To do this, shrink-wrapped μMonolayers (*n* = 3) or single cells (*n* = 2) were labeled with Vybrant DiO and 100,000 cells in 50 μL of Dulbecco’s Modified Eagle Medium (DMEM)/F12 were injected into the anterior chamber of one eye for each rabbit. The rabbits were laid on their sides with the injected eye facing down for 3 hours to allow for cell attachment and integration and then followed daily for 1 week before sacrifice and enucleation. At 1 week, the injected eyes on all five rabbits remained clear with no visible outward signs of irritation or swelling and appeared to be the same as the contralateral control eye in each animal (Fig. 6a, b, Supplementary Fig. 4). Additional examination by Confoscan indicated no abnormalities in the CE (Supplementary Fig. 5). These results establish that (i) the injection process to deliver shrink-wrapped μMonolayers to the anterior chamber does not damage the cornea, and (ii) that there is no immune response in terms of cell infiltration that would cloud the cornea due to the allogeneic rabbit CE cells.

After enucleation, the eyes were fixed, and the corneas were stained as whole mounts for the tight junctions via ZO-1 and the nuclei to observe integration into the healthy rabbit CE. Confocal microscopy imaging showed that very few DiO-labeled cells were present in the single-cell injected eyes of both rabbits (Fig. 6A and Supplementary Fig. 7a) with only a few labeled cells being found across the entire cornea. In contrast, numerous clusters of shrink-wrapped μMonolayers were integrated throughout the corneas of all 3 rabbits injected with the μMonolayers (Fig. 6b and Supplementary Fig. 7b). Higher magnification imaging showed that the cells of the shrink-wrapped μMonolayers had integrated with the healthy rabbit CE with ZO-1 present continuously at all cell borders between the DiO-labeled cells and the native rabbit CE cells (Fig. 6c). Additionally, the ECM scaffolds were still visible, providing further evidence that the shrink-wrapped μMonolayers had integrated and become a part of the rabbit CE in vivo (Figs. 6c–e).

**Fig. 7:**
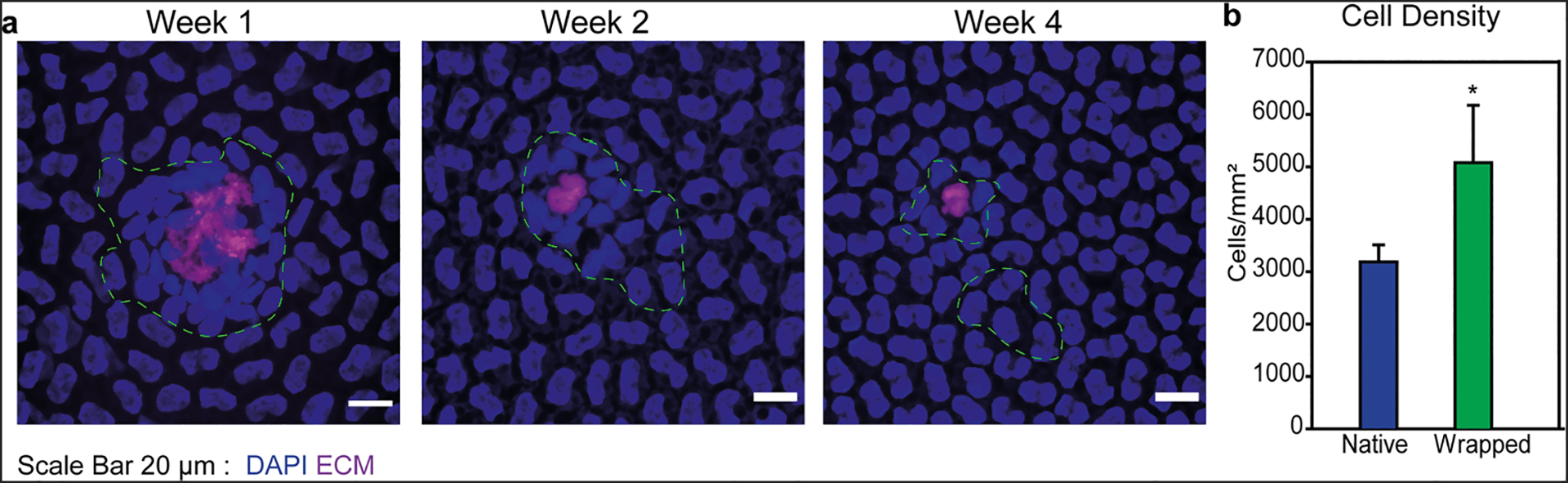
Shrink-wrapped μMonolayers exhibit stable integration into the existing healthy rabbit CE. **a** Confocal microscopy images showing the integrated shrink-wrapped μMonolayers at 1, 2-, and 4 weeks post injection. The images show that the ECM scaffold is under the cell bodies post integration and that the cell density around the ECM at 1 week post injection is higher and then begins to dissipate into the outer areas by week 4 post injection. The DiO channel has been removed for clarity and a dashed green line was drawn around the cells that were DiO positive for reference. **b** Graph showing the cell density in the areas of the integrated shrink-wrapped μMonolayers (n = 18 independent green areas, represented by the green bar) compared to native areas within the same image with no DiO-labeled cells (n = 18 independent areas with no green, represented by the blue bar). Data are represented as mean ± standard deviation and were compared using a student t test. It was found that the density in the areas with shrink-wrapped μMonolayers was significantly higher than the native CE cell density (*p < 0.05).

To determine if the shrink-wrapped μMonolayers remained stable over time and to see if the high-density areas began to spread across the cornea, we next looked at the integration of the shrink-wrapped μMonolayers at 2 weeks (*n* = 2) and 4 weeks (*n* = 3). Comparable to week 1, all eyes at weeks 2 and 4 did not have any visible signs of irritation or swelling (Supplementary Fig. 4) and Confoscan indicated no abnormalities in the endothelium (Supplementary Fig. 5). At each time point labeled CE cells and ECM squares were detected, indicating that the cells remained viable, and stably integrated. Further, the presence of the ZO-1 between the native and shrink-wrapped cells indicated that a continuous monolayer was established (Fig. 6c, d). By weeks 2 and 4, the density of the nuclei surrounding the remnant of the ECM squares decreases (Figs. 6c, d and 7a). The orthogonal views in Fig. 6e further confirm that the ECM square becomes smaller, and eventually, the remnant of the ECM square and shrink-wrapped μMonolayer cells are flush with the surrounding native CE.

Finally, we quantified the ability of the CE cells from the injected μMonolayers to stably integrate over time and increase the local cell density where they engrafted. At 1 week, cell density at locations with engrafted μMonolayers was compared to control areas within the same cornea (example areas shown in Supplementary Fig. 6). Results showed that the areas where the shrink-wrapped μMonolayers were attached and integrated had a significantly greater cell density compared to the surrounding areas of the native endothelium without shrink-wrapped μMonolayers (Fig. 7a, b). At 2- and 4-week time points, larger image areas were used as there is a possibility that the DiO fades with time due to (i) degradation of the dye and (ii) cell division that dilutes the DiO stain between two daughter cells. Therefore, we cannot be certain that all injected cells are still detectably fluorescing green. At 2 weeks, there was no significant difference in the density of the areas with DiO-labeled cells compared to areas with no labeled cells (Supplementary Fig. 8a) and one of the 4-week eyes had a statistically significant difference in the cell density of areas with labeled cells (Supplementary Fig. 8b). These results, combined with the orthogonal views from the confocal imaging (Fig. 6c–e), indicate that by 2 weeks the CE density began to equilibrate across the entire native endothelium and there were no longer the higher density patches where the μMonolayers initially engrafted. These results were consistent with the engraftment and integration of CE cells from the shrink-wrapped μMonolayer experiments performed in vitro (Fig. 4). This equilibration of the CE density across the monolayer over time is in fact the behavior that we want to see, as it shows that the CE cells delivered to the cornea appear to behave as normal CE cells. Importantly, all of the in vivo results combined show that shrink-wrapped μMonolayers were able to stably engraft, integrate into the rabbit CE, and remain viable long-term.

## Discussion

Based on our results and other research in the field, we put forward that the delivery of cells that retain their cytoskeletal structure, as opposed to single cells in solution, enhances engraftment through increased cell–cell and cell-ECM adhesions. Indeed, the clinical translation of cell injection therapy into many tissues using single cells suspended in solution where the actin cytoskeleton is largely absent has faced a number of challenges including limited cell viability, retention, and integration at the injection site^10,12,13^. In the literature, methods to improve cell injection have generally focused on increasing either the retention or adhesion of the cells at the injection location using hydrogels, small molecules in the injection media (such as the ROCK inhibitor Y-27632), pre-conditioning of cells on ECM proteins, or genetic modification^6,36,39–41^. While these methods have shown some improvements, they still fail to address the fact that single cells in solution have a rounded morphology that lacks the cytoskeletal structure and cell–cell and cell-ECM interactions of these cells in their normal tissue microenvironment. Thus, at the time of injection single cells cannot immediately interact with their environment, and must actively reestablish their cytoskeleton; cadherins, and other receptors to actively bind neighboring cells and integrins to bind the ECM. In contrast, injected cells in shrink-wrapped μMonolayers immediately start to interact with the surrounding cells and ECM as soon as they make contact, even being able to engraft into the challenging scenario of a densely packed and confluent endothelium.

For example, previous work by the Kinoshita group on the culture and injection of CE cells has highlighted the significance of the actin cytoskeleton on the adhesion of CE cells in vivo. Their research has shown that enzymatic dissociation of cells induces the phosphorylation of myosin light chain (MLC) through the Rho/ROCK pathway, which induces actin contraction and this activation of MLC negatively regulates cell adhesion^33^. To overcome this, they inject the ROCK inhibitor Y-27632 with the CE cells, which enhances cell adhesion by blocking the actin contraction, therefore increasing the interactions between the cytoskeleton, focal adhesion complexes, and integrins^36^. Cytoskeletal structure and tight junctions are necessary for CE cell function, therefore, single cells may not be able to adhere to the existing monolayer and effectively integrate. We, therefore, hypothesized that (i) injected single cells have poor viability and attachment to intact tissues due to their lack of cell–cell adhesion, cell-ECM interactions, and cytoskeletal structure and (ii) that monolayers of cells with intact cell–cell adhesions, cell-ECM interactions and cytoskeletal structure with dimensions small enough to be injected through a small gauge needle, would integrate into existing CE monolayers in higher numbers compared to single cells.

Our technology to shrink-wrap cells into μMonolayers using a thin layer of basement membrane-like ECM uniquely maintains high cell viability, cell–cell junctions, cell-ECM binding, and cytoskeletal structure post injection. As shown, using the CE as a model system these μMonolayers engrafted within a few hours, established tight junctions, and formed an organized F-actin cytoskeletal structure. When compared to enzymatically-passaged single cells, injected shrink-wrapped μMonolayers engrafted at significantly higher numbers and increased CE monolayer cell density in vitro. Importantly, shrink-wrapped μMonolayers showed a high rate of engraftment into in vitro engineered low-density monolayers, ex vivo corneas, and in vivo corneas. This was achieved without the need to disrupt cell–cell tight junctions or remove the existing cells prior to seeding, which is the standard clinical practice for those patients with remaining CE cells^6^. The in vivo rabbit studies were performed utilizing healthy rabbit eyes and results showed high numbers of shrink-wrapped μMonolayers integrated into the healthy CE and remained integrated over 4 weeks. This is extremely promising as cells within a young healthy rabbit endothelium are contact-inhibited, have tight junctions, and are at an extremely high density. Therefore, if the shrink-wrapped μMonolayers can integrate within such a tissue, integration into damaged or diseased CEs with a much lower cell density should occur at much higher rates. This is further evidence that injection of μMonolayers could be used for patients who are experiencing declines in cell density and visual acuity in order to boost their CE cell density before it reaches the lower limit where corneal blindness results. This would increase the lifespan of their existing cornea and could eliminate the need for a future transplant.

The results of this study are extremely promising, particularly because in vivo CE cells integrated into a healthy, high-density endothelium that likely represents the most challenging engraftment environment. This is because CE cell density in the healthy rabbit cornea is ~3000 cells/mm^2^, which is very high, and it is encouraging that at sites of engraftment μMonolayers transiently increased the density to ~5000 cells/mm^2^ before migrating out over time (Fig. 7 and Supplementary Fig. 8). Ideally, this technology would be tested in an in vivo model that is representative of patients with a low CE cell density of <500 cells/mm^2^, our target population. However, such an in vivo model does not currently exist^42^, as the low-density observed in humans is related to genetic predisposition, aging, and exposure to UV light^43,44^. Developing an animal model to effectively recapitulate this human disease state is thus challenging. For these reasons, we chose the healthy rabbit as an in vivo model because it was more relevant than a complete injury and removal model that has been used in other rabbit CE studies. There are limitations, however, such as the variability in cell density between corneas, even from the same rabbit. This adds a layer of difficulty to quantifying any effect on cell density within the healthy CE model. However, the results from our in vitro studies utilizing lower density CE monolayers showed a significant increase in cell density of >50% for μMonolayers compared to single-cell controls (Fig. 4). This result, combined with the in vivo rabbit studies (Figs. 6 and 7), demonstrates shrink-wrapped CE cell μMonolayers can engraft into endothelial tissues at a high level.

As we take the next steps towards clinical translation, we will expand upon these proof-of-concept studies to include larger in vivo rabbit studies in both healthy and fully-stripped CE models. While these do not fully represent patients with low CE cell density, the fully-stripped endothelium model is in line with how CE cell replacement using single cells is done in current human clinical trials and will allow us to compare this technology more directly with those methods. We also hope to expand this technology to cell delivery in other organs that suffer from donor shortages, such as the heart and liver where we could restore function through cardiomyocyte and hepatocyte delivery, respectively. There are also diseases where shrink-wrapped μMonolayers engrafted into epithelial layers could provide new therapeutic options, such as the delivery of airway cells genetically edited to restore cystic fibrosis transmembrane conductance regulator (CFTR) function for cystic fibrosis patients, or delivering alveolar type II epithelial cells to damaged distal regions post COVID-19 infection. While there are many paths toward clinical impact, the key take-home of this work is that it is possible to use the shrink-wrapping approach to engineer small clusters of cells that retain key phenotypic markers and are primed for rapid and enhanced engraftment into tissues, compared to existing alternatives.

## Materials and methods

### Study design.

The research objectives of this study were to: (1) adapt our previously published technique to shrink-wrap single cells in order to shrink-wrap small clusters of CE cells into patches of endothelium termed μMonolayers, (2) to investigate whether shrink-wrapped μMonolayers would integrate into endothelium and increase cell density in vitro, and (3) determine if the μMonolayers were capable of engrafting into an existing healthy endothelium in vivo. Primary bovine and rabbit CE cells were used throughout the study using established cell culture methods, with minor modifications in the case of the rabbit cells26,36. Sample sizes for in vitro studies were determined by using the minimum number of samples to be considered statistically significant and time points/endpoints were based on our previously published studies. For the in vitro cell density study, 4 replicates per sample type per time point were used and one full study was completed. Data from the day 3-time point was used to determine if the sample size was sufficient to provide statistical significance. On day 7 one control sample and on day 14 one single cells sample was lost during fixing and staining and therefore *n* = 3 for those sample types. In vivo studies were designed to be pilot studies and as such, the number of rabbits per study was kept to 2–3 animals per time point and condition to establish repeatability.

### ECM scaffold fabrication.

The ECM scaffolds were fabricated via previously described surface-initiated assembly techniques with minor modifications24,25. Briefly, 1 cm × 1 cm PDMS stamps designed to have 200 μm square features were fabricated via standard soft lithography techniques. The stamps were sonicated in 50% ethanol for 60 minutes, dried under a stream of nitrogen, and incubated for 60 minutes with a 50:50 mixture of 50 μg/mL COL4 and 50 μg/mL LAM (Fig. 1 **step 1**). Either 50% AlexaFluor 488 labeled COL4 or 50% AlexaFluor 633 labeled LAM (for a final concentration of 25% labeled protein) was used to visualize the pattern transfer. Following incubation, the stamps were rinsed in sterile water, dried under a stream of nitrogen, and brought into conformal contact with PIPAAm (2% high molecular weight, Scientific Polymers) coated 18 or 25 mm glass coverslips for 30 minutes to ensure transfer of the squares (Fig. 1 **step 2**). ECM squares microcontact printed on PDMS coverslips were used as controls. Upon stamp removal, laser scanning confocal microscopy (Nikon AZ100) was used to determine the quality of the transferred ECM squares.

### Corneal endothelial cell culture.

Bovine CE cells were isolated and cultured as previously described26,27. Briefly, corneas were excised from the whole globe (Pel Freez Biologicals) and incubated endothelial side up in a ceramic 12-well spot plate with 400 μL of TrypLE Express for 20 minutes. The cells were then gently scraped from the cornea using a rubber spatula, centrifuged at 1500 rpm for 5 minutes, resuspended in 5 mL of culture media (low glucose DMEM with 10% FBS, 1% Pen/Strep/AmphB, and 0.5% gentamicin), designated at P0 and cultured in a 50 kPa PDMS coated T-25 flask that was pre-coated with COL426. Fifty whole eyes were received at a time and were used to seed 5 T-25 flasks. Cells were cultured until confluence and split 1:3 until they were used once confluent at P2.

Rabbit corneas were excised from the whole globe (Pel Freez Biologicals), the CE and Descemet’s Membrane were manually stripped with forceps, and then incubated in Dispase (1 U/mL, Stem Cell Technologies) for 1.5 hours at 37 °C to detach the rabbit CE cells (RCECs) from the Descemet’s Membrane. The RCECs were then gently pipetted up and down, diluted in culture media (DMEM/F12, 10% FBS, 0.5% Pen/strep), centrifuged at 1500 rpm for 5 minutes, resuspended in 10 mL of culture media, designated at P0 and cultured on COL4 coated T-25 flasks with the equivalent of 15–25 eyes per flask depending on cell yield. RCECs were cultured until confluence and then split 1:2 and used in all experiments once confluent at P1 or P2.

### Shrink-wrapping CE Cell μMonolayers in ECM.

Patterned coverslips were secured with vacuum grease to the bottom of 35 mm Petri dishes, which were placed on a dry block set to 52 °C. This resulted in the coverslips reaching (within 30 min) and holding at ~40 °C. Bovine CE cells were released from the culture flask with TrypLE Express, centrifuged, and resuspended at a density of 150,000 cells/mL in 15 mL centrifuge tubes. The tubes were placed in a dry block set at 45 °C for ~5 minutes, or until the cell solution just reached ~40 °C and 2 mL of cell suspension was added to each 35 mm dish before it was immediately placed in an incubator (37 °C, 5% CO_2_). Cells were cultured for 24 hours to allow them to form μMonolayers on the 200 μm ECM squares. Samples were then removed from the incubator, rinsed twice in 37 °C media to remove non-adherent single cells, 2 mL of fresh warm media was added, and the sample was allowed to cool to room temperature. Once the temperature decreased to <32 °C the PIPAAm dissolved and released the μMonolayers. The release process was recorded using a Photometrics CoolSnap camera. Following the release, the μMonolayers were collected via centrifugation at 1500 rpm for 5 minutes before use in further experiments. CE cells seeded onto PDMS coverslips were used as a control.

### Immunostaining of shrink-wrapped CE cell μMonolayers.

Shrink-wrapped bovine μMonolayers resuspended in PBS containing Ca2+ and Mg2+ (PBS++) were injected through a 28-gauge needle onto a glass coverslip and allowed to settle for ~15 minutes before fixation for 15 minutes in 4% paraformaldehyde in PBS++. Samples were gently washed two times with PBS++ and incubated with 1:100 dilution of DAPI, 1:100 dilution of mouse anti-ZO-1 antibody (Life Technologies), and 3:200 dilution of AlexaFluor 488. Samples were rinsed two times for 5 minutes with PBS++ and incubated with 1:100 dilution of AlexaFluor 555 goat anti-mouse secondary antibody for 2 hours. Samples were rinsed two times for 5 minutes with PBS++, mounted on glass slides with Pro-Long Gold Antifade (Life Technologies), and then imaged on a Zeiss LSM700 confocal microscope.

### Viability of shrink-wrapped CE cells μMonolayers post injection.

After centrifugation, bovine shrink-wrapped μMonolayers or single cells were resuspended in 200 μL of growth media, drawn up into a 28 G needle, injected into a petri dish, and incubated with 2 μM calcein AM and 4 μM EthD-1 (Live/Dead Viability/Cytotoxicity Kit, Life Technologies) in PBS++ for 30 minutes at 37 °C. After 30 minutes, samples were imaged on a Zeiss LSM700 confocal; 5 images per sample and 3 samples per type were used. The number of live and dead cells was counted manually using ImageJ’s multi-point tool. The number of live cells was divided by the number of total cells to determine the percent viability of both the shrink-wrapped cells and enzymatically released cells. The data were compared using a Student’s *t* test in SigmaPlot. The same methods were used to test the viability of the cells through a 34 G needle to test the smallest needle that could be used.

### Seeding of shrink-wrapped CE cell μMonolayers and single CE cells on stromal mimics.

Compressed collagen type I films were prepared as previously described to mimic the structure of the underlying stroma27. Briefly, a 6 mg/mL collagen type I gel solution was prepared per the manufacturer’s instructions and pipetted into 9 mm diameter silicone ring molds on top of glass coverslips. The gels were placed into a humid incubator (37 °C, 5% CO_2_) for 3 hours to compress under their own weight. The gels were then dried completely in a biohood followed by rehydration in PBS, forming thin collagen type I stromal mimic of approximately 10 μm in thickness (reduced from an original 1 mm in thickness as cast). Shrink-wrapped bovine CE cell μMonolayers were seeded onto the films at a 1:1 ratio of the stamped coverslip to collagen type I film. As a control, bovine CE cells that were cultured in the flasks and enzymatically released using TrypLE Express into a single-cell suspension were seeded onto collagen type I films. The number of control single cells seeded was equal to the number of cells seeded in the μMonolayers, assuming each of the 200 μm ECM squares used for shrink-wrapping was completely covered in cells. The average number of cells on the 200 μm ECM squares was 30 cells, so with 1600 squares per stamp, we seeded ~48,000 cells per sample. Therefore, 50,000 cells per sample were seeded for the controls. At 6 and 24 hours, samples were removed from the culture and fixed and stained for the nucleus, ZO-1 (tight junction protein), and F-actin. Briefly, samples were rinsed two times in PBS++, fixed in 4% paraformaldehyde in PBS++ with 0.05% Triton-X 100 for 15 minutes. Samples were rinsed two times for 5 minutes with PBS++ and incubated with five drops of NucBlue (Life Technologies) for 10 minutes. Samples were rinsed once with PBS++ and incubated with 1:100 dilution of mouse anti-ZO-1 antibody (Life Technologies) and 3:200 dilution of AlexaFluor 488 or 633 phalloidin for 2 hours. Samples were rinsed three times for 5 minutes with PBS++ and incubated with 1:100 dilution of AlexaFluor 555 goat anti-mouse secondary antibody for 2 hours. Samples were rinsed three times for 5 minutes with PBS++, mounted on glass slides using Pro-Long Gold Antifade, and imaged on a Zeiss LSM700 confocal microscope.

### In vitro integration of shrink-wrapped μMonolayers vs single bovine CE cells.

To mimic a low-density CE, 25,000 P5 bovine cells were seeded onto the collagen type I stromal mimics, as described above, until confluent to form the low-density monolayers. Shrink-wrapped bovine μMonolayers and single bovine CE cells were prepared as above, labeled with CellTracker Green (Life Technologies) for 30 minutes, centrifuged, diluted to the equivalent of 50,000 cells/sample, and injected onto the low-density monolayers. Low-density monolayers with no cells injected on top served as controls. Samples were rinsed 3 hours post injection to mimic the in vivo procedures and new media was added. The media was changed every two days thereafter. Samples were fixed and stained on days 3, 7, and 14 as described above. A Zeiss LSM700 confocal was used to image 10 random spots on each sample and the cell density was manually counted using the multi-point selection tool in ImageJ to count cell nuclei. The locations on each sample were chosen randomly using the joystick positioner on the Zeiss confocal without looking at the sample under the eye piece to ensure a random location within each sample. The number of nuclei was divided by the image area to obtain the cells/mm2 per image. The cell density for each sample was determined by averaging the cell densities of each image and the average cell density of each sample type was determined by averaging the cell density of the 3–4 samples. The data were compared using a one-way ANOVA on ranks with Tukey’s test (day 3) or one-way ANOVA (days 7 and 14) with Tukey’s test in SigmaPlot. To examine the outgrowth of the shrink-wrapped μMonolayers over time, confocal images centered around an individually shrink-wrapped μMonolayer (day 3 *n* = 33, day 7 *n* = 37, day 14 *n* = 40) were collected and the CellTracker channel was converted into a binary black and white image. The binary images for each sample type were then converted into one Z-stack and analyzed via the Heat Map for Z-stacks plugin (relative without log10) for ImageJ to determine the average pixel density of CellTracker. To determine the percentage of injected single cells or shrink-wrapped cells that were engrafted into the existing monolayers at each time point, the average number of cells on the control samples was subtracted from the total number of cells on the single-cell or shrink-wrapped samples to get the total number of cells that engrafted using each technique. This difference in the number of cells was then divided by the number of seeded cells (~50,000) and multiplied by 100 to achieve a percentage. The results were compared at each time point for the two sample types using a student’s *t* test *p* < 0.05.

### Live imaging of in vitro integration of shrink-wrapped bovine CE cells.

For live imaging, the bovine monolayer on the collagen type I stromal mimic was first incubated for 30 minutes with CellTracker Orange to differentiate between the existing monolayer and injected cells, which were labeled with CellTracker Green as described above. HEPES buffered Opti-MEM I Reduced Serum Media (Life Technologies) with 10% FBS, 1% Pen/Strep was added to the monolayer and shrink-wrapped bovine μMonolayers that were prepared as described above were injected through a 30 G needle on top of the sample. The sample was placed on the Zeiss LSM700 confocal equipped with a temperature chamber set to 37 °C for 30 minutes to allow for the cells to settle. Using the Definite Focus system, a time-lapse series of one z-stack was obtained every hour for 48 hours. Videos from the time-lapse images were created using the Imaris Software.

### Ex vivo integration of shrink-wrapped rabbit CE cells.

Whole rabbit eyes were placed cornea up in a 12-well plate and shrink-wrapped rabbit CE μMonolayers were prepared as described above. Two samples of μMonolayers per ex vivo eye were prepared and resuspended in 100 μL of DMEM/F12. A 30-G insulin syringe was used to draw up the full 100 μL, the needle was inserted into the center of the cornea until it was visible in the anterior chamber and 50 μL of the suspension was injected. This resulted in the equivalent of 50,000 cells injected into the anterior chamber. The needle was held in place for a few seconds to ensure the media and cells did not come back out of the injection site. The injection was viewed under a stereomicroscope and the pink color of the media filling the anterior chamber was visible, indicating successful injection. The whole eyes were flipped and incubated cornea down for 3 hours at 37 °C, 5% CO_2_ in a humidified incubator. No media was added directly to the eyes, instead, media was included in two empty wells to maintain hydration throughout the study (Supplementary Fig. 3). After 3 hours, the whole eye was placed in 2% paraformaldehyde (PBS++) at 4 °C for 24 hours. After 24 hours the eye was rinsed in PBS and the cornea was excised and rinsed three times for 5 minutes. The cornea was then incubated CE facing down on 1 mL of PBS++ containing 2 drops of NucBlue (Life Technologies), 2:100 dilution of mouse anti-ZO-1 antibody (Life Technologies), and 3:200 dilution of AlexaFluor 488 Phalloidin (Life Technologies) for 2 hours at room temperature. Corneas were then rinsed three times for 5 minutes in PBS followed by a 2-hour incubation in 1 mL PBS++ with 2:100 dilution of AlexaFluor 555 goat anti-mouse secondary antibody for 2 hours and stored in PBS before imaging on the Zeiss LSM700 confocal.

### In vivo injection and integration of shrink-wrapped CE cells.

All experimental procedures were reviewed and approved by the University of Pittsburgh Institutional Animal Care and Use Committee (IACUC) and carried out according to the ethical guidelines of the Association for Research in Vision and Ophthalmology Resolution on the Use of Animals in Ophthalmic and Vision Research. For both in vivo experiments, shrink-wrapped rabbit μMonolayers were prepared as described above with one minor modification: cells were labeled with Vybrant DiO 1 day prior to seeding onto the ECM nano-scaffolds by incubating cells in 1 mL of media with 5 μL of Vybrant DiO for 30 minutes followed by three, 10-minute rinses with fresh media. An excess number of μMonolayer samples were prepared to ensure there was enough volume for injection. The shrink-wrapped μMonolayers were released as described above and after centrifugation at 1500 rpm for 5 min, the shrink-wrapped μMonolayers were resuspended in DMEM/F12 at the equivalent of 100,000 cells per 50 μL injection volume (2 stamped samples per 50 μL).

For the first experiment, control single cells were prepared as described above and resuspended in DMEM/F12 at a density of 100,000 cells in a 50 μL injection volume. Six female New Zealand white rabbits with healthy intact CEs weighing approximately 2.5 kg were used for this study. Rabbits were anesthetized with Ketamine (40 mg/kg) and Xylazine (4 mg/kg) intramuscular injection followed by isoflurane inhalation to keep rabbits under sedation for 3 hours. One rabbit did not survive the anesthetization. Rabbits #1 & 2 were injected in the right eye with 50 μL (~100,000 cells) of the single-cell suspension. Rabbits #3, 4, and 5 were injected with 50 μL of the shrink-wrapped μMonolayer suspension into the right eye using a 30 G needle attached to a 500 μL syringe. A tunnel in the corneal stroma was made for the injecting which prevented cell leakage after injection. Immediately after injection, each rabbit was placed on its side with the injected eye facing down for 3 hours to ensure attachment of the cells. On day 7, rabbits were anesthetized with an intramuscular injection of ketamine (40 mg/kg) and xylazine (4 mg/kg) and then euthanized with Euthasol solution (1 mg per 4 lbs) containing (390 mg/mL Sodium Pentobarbitol, 50 mg/mL Phenytoin Sodium) through an ear vein injection. Photographic images were obtained via the Google Pixel 2 camera (Supplementary Fig. 4) to document eye clarity and the endothelium was viewed using a Nidek Confoscan 3 (Supplementary Fig. 5). Eyes were then immediately enucleated and intravitreally injected with 100 μL 2% paraformaldehyde in PBS++. The whole eye was then immersed in 2% paraformaldehyde in PBS++ and fixed at 4 °C for 24 hours. After 24 hours the eye was rinsed in PBS and the cornea was excised and rinsed 3x’s for 5 minutes. The cornea was then incubated CE facing down on 1 mL of PBS++ containing two drops of NucBlue (Life Technologies) and 2:100 dilution of mouse anti-ZO-1 antibody (Life Technologies) for 2 hours at room temperature. Corneas were then rinsed three times for 5 minutes in PBS followed by 2-hour incubation in 1 mL PBS++ with 2:100 dilution of AlexaFluor 555 goat anti-mouse secondary antibody for 2 hours and stored in PBS before imaging on the Zeiss LSM700 confocal or a Nikon FN1 base with an A1R HD MP Confocal module. To quantify the density of the integrated cells, the green cells were manually traced with the freehand selection tool in ImageJ, and the “Measure” function was used to determine the area. The multi-point selection tool was used to determine the number of nuclei within that area and the density was determined by dividing the number of nuclei by the area. The rectangle tool was used to select control areas, areas of nongreen (i.e., non-DiO-labeled) cells, and elsewhere in the image of a similar area. The area and cell number were determined the same way as for the DiO-labeled areas and the cell density was calculated by dividing the number of nuclei by the area. The number of control areas per image was matched to the number of DiO-labeled areas (Supplementary Fig. 6). Data from the injected eye of both rabbits were pooled together and the cell density of the areas with green cells were statistically compared to those without using SigmaPlot for a student *t* test.

For the second experiment, only shrink-wrapped μMonolayers were used to determine if cells would integrate and remain in the CE long-term. Six female New Zealand white rabbits with healthy intact CEs weighing approximately 2.5 kg were used for this study. Because these were initial proof-of-concept studies, we wanted to eliminate any possible sex differences and included only female rabbits. Rabbits were anesthetized as described above and injected with 50 μL of the shrink-wrapped μMonolayer suspension into the right eye. Immediately after injection, each rabbit was placed on its side with the injected eye facing down for 3 hours to ensure attachment of the cells. One rabbit went into tachycardia right at the end of the 3 hours and was revived, however, it suffered brain damage as a result of the amount of time without oxygen. That rabbit was sacrificed 24 hours post injection and the eye was removed and processed as described above and used to determine if the injection processed had been successful (data not shown). At 14 days (2 rabbits) or 28 days (3 rabbits) post injection, rabbits were sacrificed (as described above) and photographic images were obtained via the Google Pixel 2 camera (Supplementary Fig. 4) to document eye clarity and the endothelium was viewed using a Nidek Confoscan 3 (Supplementary Fig. 5). Following imaging, eyes were immediately enucleated and fixed, stained, and stored as described above, before imaging on the Zeiss LSM700 confocal or a Nikon FN1 base with an A1R HD MP Confocal module. To quantify the cell density, images with DiO-labeled cells present were taken (*n* = 10+ images) and images were taken far away in areas where there were no green cells (*n* = 5 images) in each cornea. Because the DiO-labeled may have faded over time, in this case, the cell density of the entire image was counted using the number of nuclei (counted manually via the multi-point tool with only full nuclei being counted) divided by the area of the image. To statistically compare the data, for each rabbit, the density of the images with green cells was compared to the density of the images with no green cells in SigmaPlot using the student *t* test.

## Supplementary Material

Video 1

Video 2

Video 3

Video Captions

Supplemental Information

## Data Availability

All raw data are available upon written request to the authors. Anyone requesting materials related to shrink-wrapping cells must complete an MTA in order to receive those materials.
