# Supplementary material for: In vivo engraftment into the cornea endothelium using extracellular matrix shrink-wrapped cells
Source: Commun Mater. Author manuscript; Available in PMC 2024 Aug 22. (PMC11340414; doi:10.1038/s43246-022-00247-1)
Supplement: Video Captions [file NIHMS1920012-supplement-Video_Captions.pdf]

Description of Additional Supplementary Files

File Name: Supplementary Video 1

Description: Release of Shrink-wrapped µMonolayers. A time-lapse video showing the release of the shrink-wrapped µMonolayers. Warm PBS with calcium and magnesium was added to the sample and as the temperature decreases below the LCST of PIPAAm, it dissolves, resulting in the release and shrink-wrapping of the µMonolayers.

File Name: Supplementary Video 2

Description: Time-lapse In Vitro Integration of Shrink-wrapped µMonolayers (Top-down view). This is a time-lapse confocal microscopy video from the top-down view showing the integration of the shrink-wrapped µMonolayers (labeled with CellTracker Green) into an existing monolayer of CE cells (labeled with CellTracker Orange, appearing RED). The micropatterned ECM used to shrink-wrap the µMonolayers is shown in purple.

File Name: Supplementary Video 3

Description: Time-lapse In Vitro Integration of Shrink-wrapped µMonolayers (Side view). This is a time-lapse confocal microscopy video rendered from the side view showing the integration of the shrink-wrapped µMonolayers (labeled with CellTracker Green) into an existing monolayer of CE cells (labeled with CellTracker Orange, appearing RED). The micropatterned ECM used to shrink-wrap the µMonolayers is shown in purple.
yers is shown in purple.
