# Supplementary material for: In vivo engraftment into the cornea endothelium using extracellular matrix shrink-wrapped cells
Source: Commun Mater. Author manuscript; Available in PMC 2024 Aug 22. (PMC11340414; doi:10.1038/s43246-022-00247-1)
Supplement: Supplemental Information [file NIHMS1920012-supplement-Supplemental_Information.pdf]

Supplemental Information:

Injury-Free In Vivo Delivery and Engraftment into the Cornea Endothelium Using Extracellular Matrix Shrink-Wrapped Cells

Rachelle N. Palchesko[1]*, Yiqin Du[2], Moira L. Geary[2], Santiago Carrasquilla[1], Daniel J. Shiwarski[1], Irona Khandaker[2], James L. Funderburgh[2], Adam W. Feinberg[1,3]*

Affiliations:

[1]Department of Biomedical Engineering, Carnegie Mellon University, 5000 Forbes Avenue, Pittsburgh PA, 15213.

[2]Department of Ophthalmology, University of Pittsburgh, 203 Lothrop Street, Pittsburgh PA, 15213.

[3]Department of Materials Science & Engineering, Carnegie Mellon University, 5000 Forbes Avenue, Pittsburgh PA, 15213.

*To whom correspondence should be addressed: rachelle@andrew.cmu.edu (R.P.); feinberg@andrew.cmu.edu (A.W.F.)

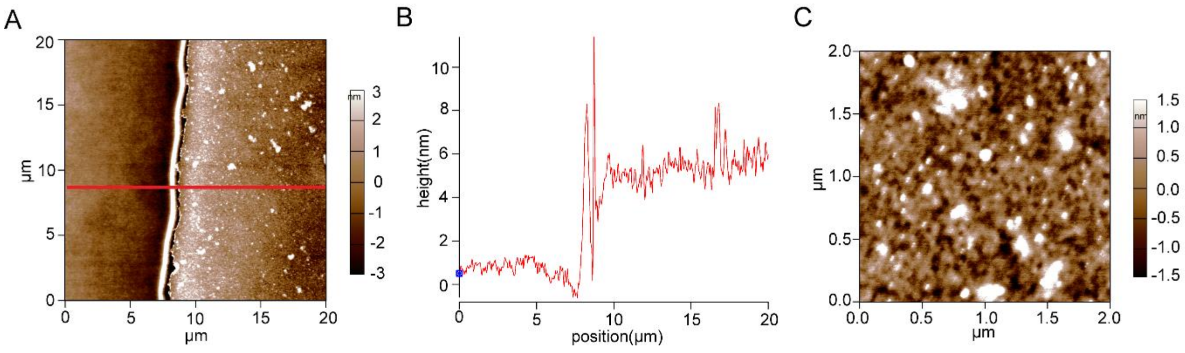

Supplementary Figure 1. AFM images showing nanostructure and height of the patterned ECM nanoscaffolds. (A) AFM scan of the side of an ECM nanoscaffold. (B) Height trace of the red line shown in A showing that at the very edge the scaffold is approximately 10 nm high, but that the scaffold is between 4-6 nm across the rest of the scaffold. (C) Zoomed in AFM scan of the patterned ECM showing the basement membrane like structure of the scaffold. Images were acquired on an Asylum Research MFP-3D-BIO AFM in AC in air mode using AC-160 cantilevers.

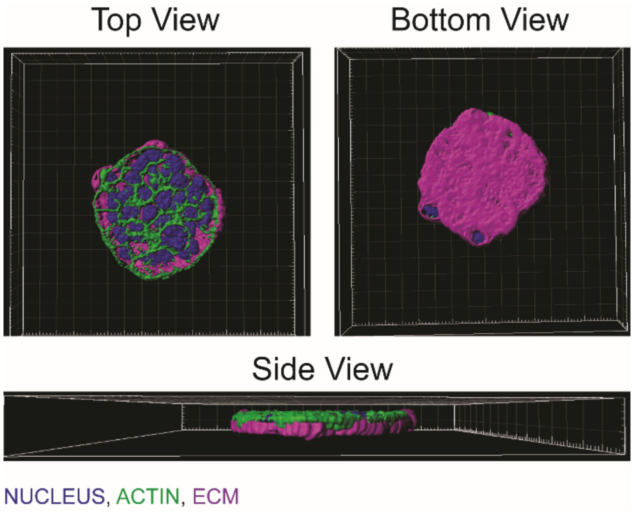

Supplementary Figure 2: 3D rendering of confocal microscopy images showing the ECM coverage on the μMonolayers. The panel on the left shows the top-down view of a μMonolayer, with the nuclei shown in blue, the F-actin in green and the ECM is in purple. The ECM appears to be under the cells. The right panel is a bottom view (180 degree flip in the z-direction) of the same μMonolayer, showing the complete coverage of the bottom of the cells in the ECM scaffold. The side view confirms that the ECM is on the bottom side of the cells only.

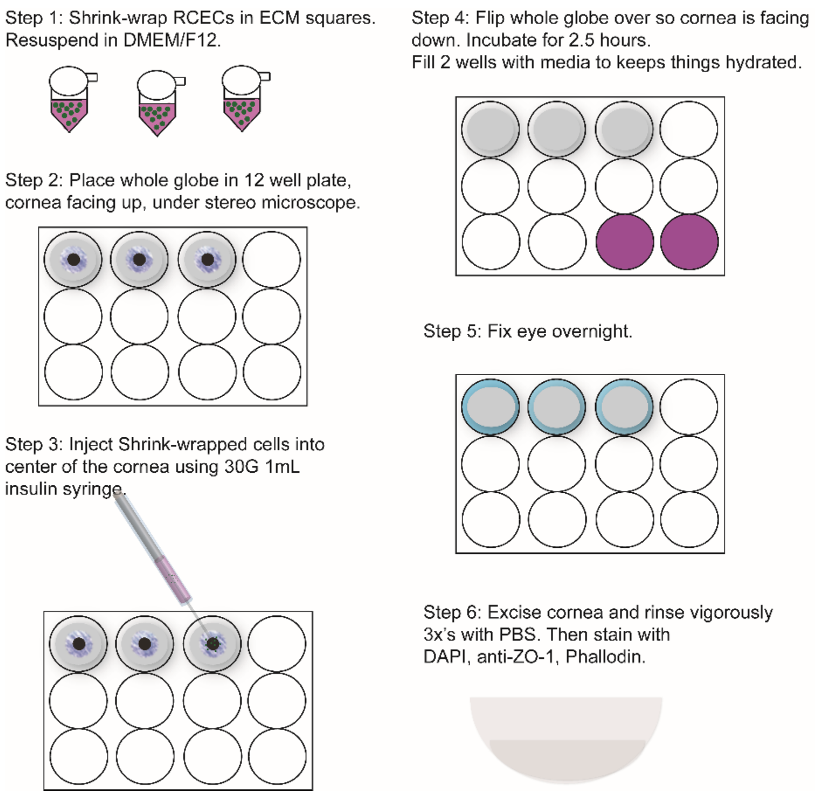

Supplementary Figure 3: Schematic showing the ex vivo experimental setup. In Step 1, shrink-wrapped rabbit CE cells (RCECs) were suspended in sterile DMEM/F12 media. In Step 2, Whole enucleated rabbit eyes (i.e., globes) were placed cornea side up in a 12 well plate. In Step 3, the shrink-wrapped RCECs from Step 1 were injected into the anterior chamber to simulate the clinical process of cell injection for CE repair. In Step 4, the globes were flipped upside down with the cornea facing the bottom of the well. This was done so that the injected shrink-wrapped cells would contact the posterior surface of the cornea through gravitational settlement. In Step 5, the globes were fixed in paraformaldehyde overnight. In Step 6, the corneas were cut out from the globes, rinsed thoroughly, and then immunofluorescently stained for nuclei, ZO-1, and F-actin.

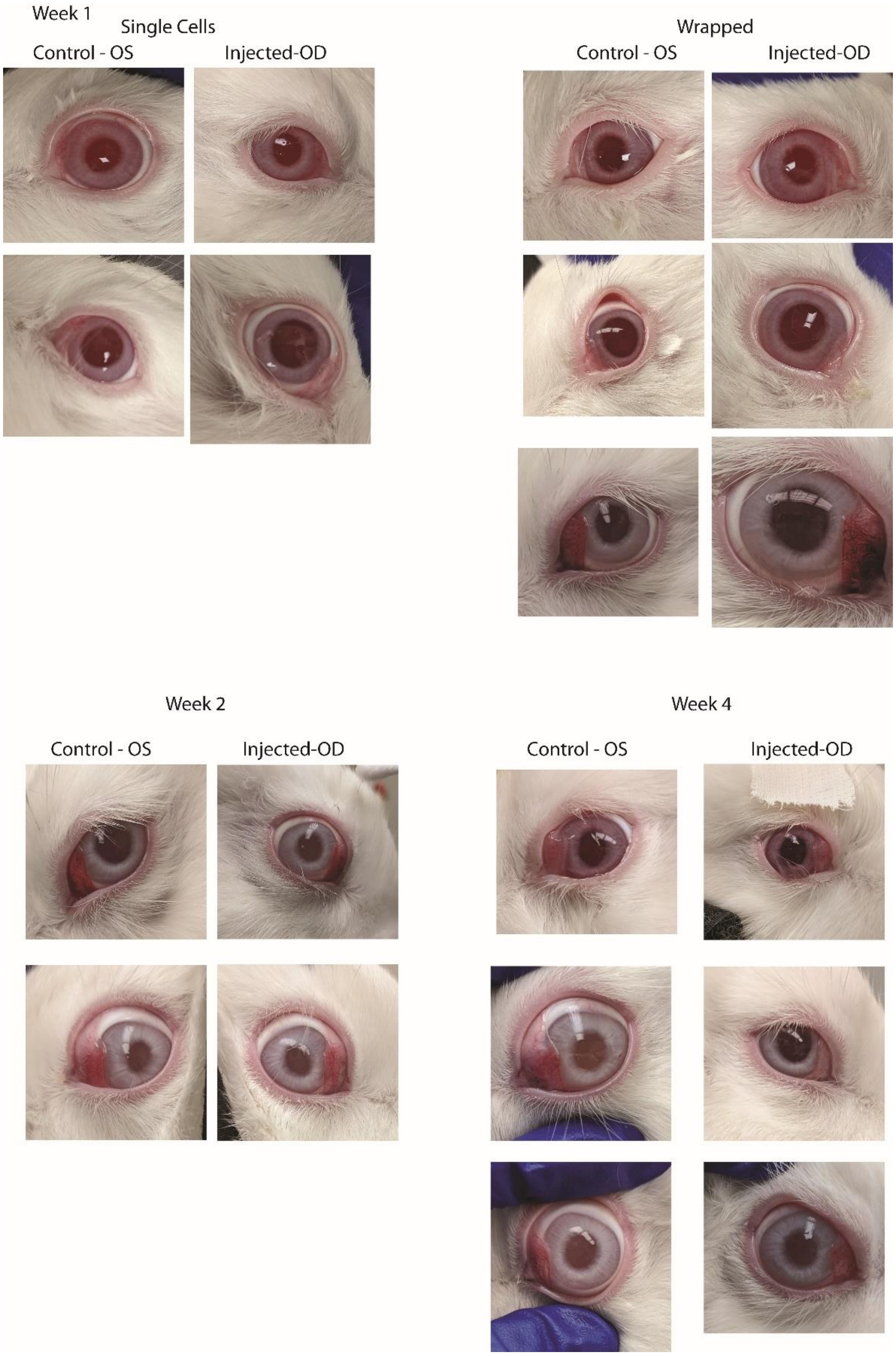

Supplementary Figure 4: Rabbit eye photographs. Images showing the uninjected control eye (OS) of each rabbit, compared to the injected (OD) eye of each rabbit. All eyes were clear at all time points showing no cloudiness or obvious irritation from the injection of single or shrink-wrapped μMonolayer cells.

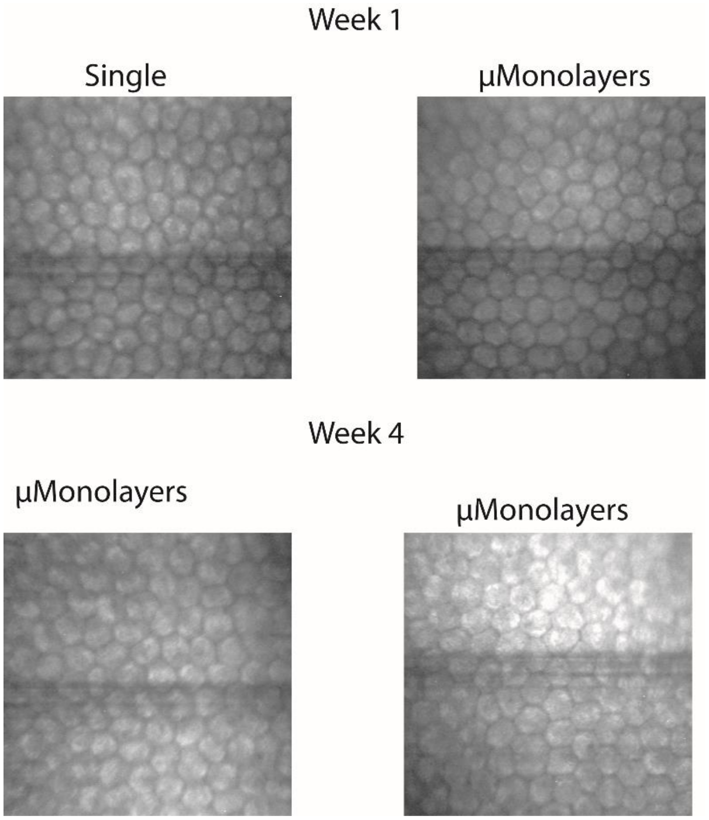

Supplementary Figure 5: Confoscan showing normal cobblestone morphology of corneas post injection at 1 and 4 weeks. Rabbit eyes injected with DiO labeled endothelial cells, either single cells or shrink-wrapped as µMonolayers, were imaged with the Confoscan at 1 and 4 weeks. The endothelium appeared normal, with the expected cobblestone morphology and cell density.

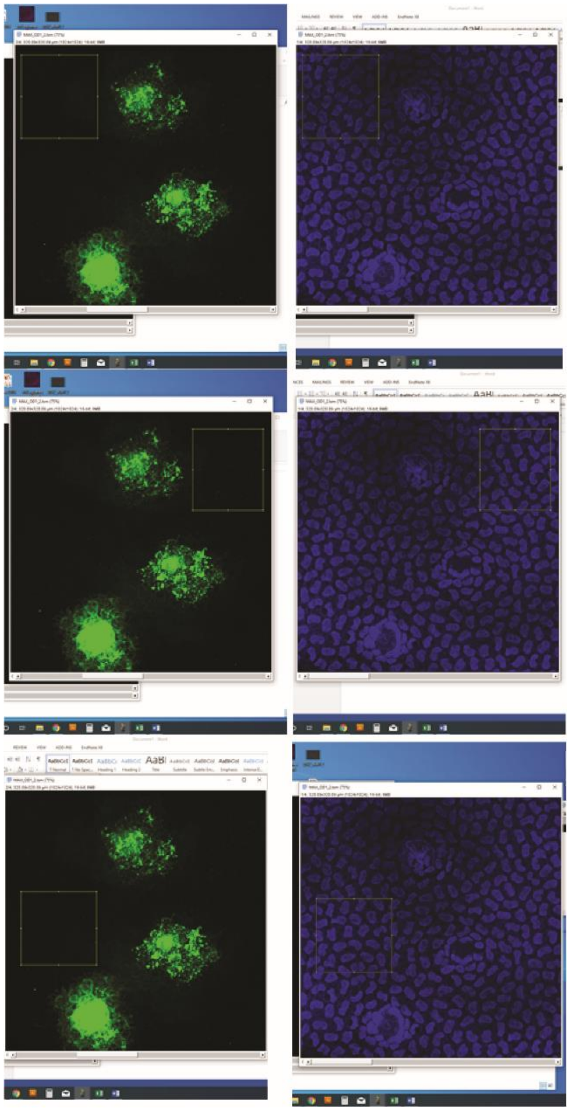

Supplementary Figure 6: Examples of equivalent areas used for cell density calculations. For the DiO labled areas, the cells were traced with the pen feature in ImageJ, the nuclei were manually counted and the area was determined by ImageJ. To get the native density the areas shown above in the boxes in yellow were used. The same number of equivalent areas as DiO labeled areas were counted per image and the areas were designed to be approximately the same size as the DiO labeled regions.

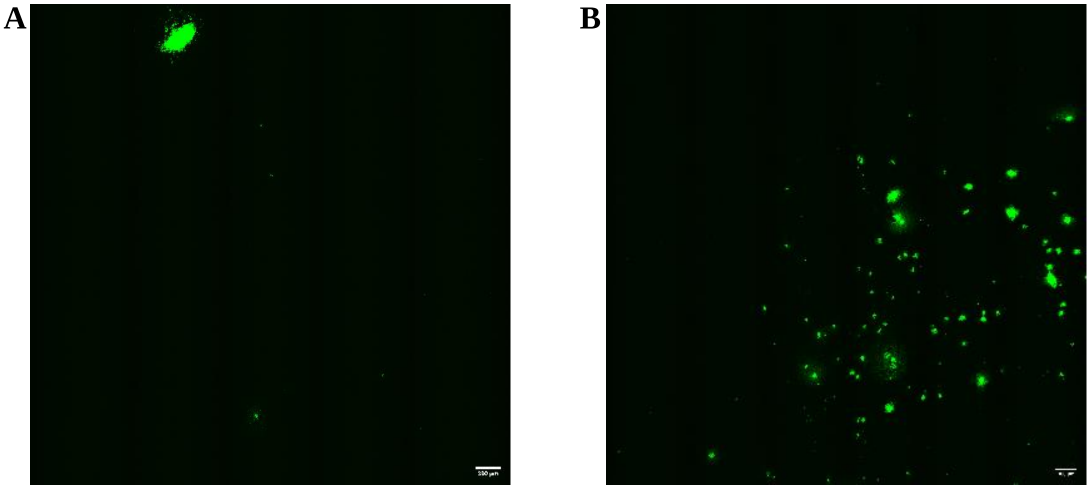

Supplementary Figure 7: Large area Tile-scans of In Vivo Corneas 1 week Post-Injection. (A) A representative image of the cornea with single cells injected (5 X 5 mm tile scan). The large spot is the area of the injection and some green signal is due to light reflection from the damage to the stroma. (B) A representative image of DiO positive µMonolayers present in the corneal endothelium 1 week post-injection (5 X 5 mm tile scan). Scale bars = 300 µm

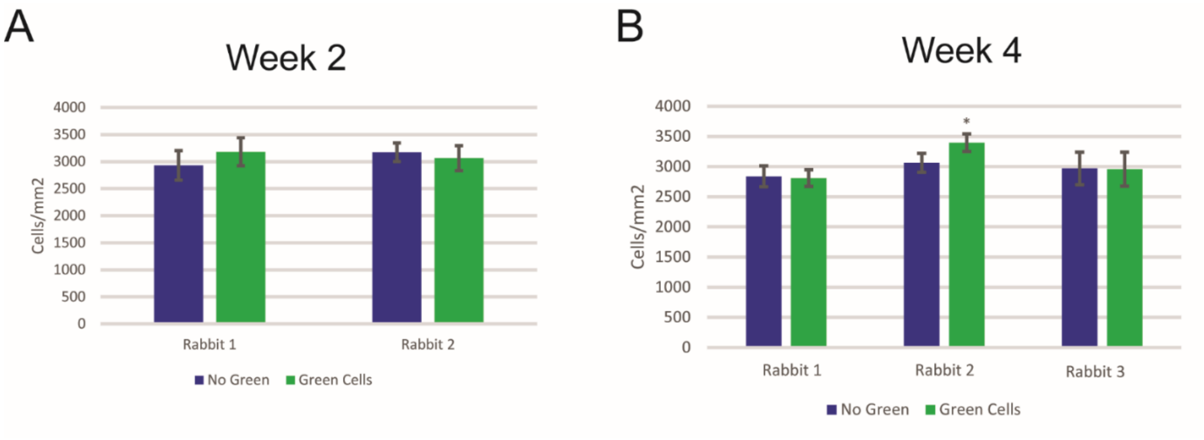

Supplementary Figure 8: Cell density of areas with green DiO labeled cells compared to areas with no labeled cells. To quantify the cell density of the rabbit CEs post injection, images in areas with DiO labeled cells present were taken (n=10+ images) and images were taken far away in areas where there were no green cells (n=5 images) in each injected cornea. Because the DiO labeled may have faded over time, in this case the cell density of the entire image was counted using the number of nuclei (counted manually via the multi-point tool with only full nuclei being counted) divided by the area of the image. To statistically compare the data, for each rabbit cornea, the average density of the images with green cells was compared to the average density of the images with no green cells in SigmaPlot using student T-test. Data is represented as mean ± standard deviation and * indicates statistically significant $p<0.05$.
